# The Medical Profession

**Published:** 1843-11

**Authors:** 


*The Medical Profession.*—At a period of political excitement, when the minds of men are sharpened against each other by ever-recurring subjects of contention; when intolerance and proscription usurp the name of patriotism, and individual opinion is borne down and trampled upon by irresponsible masses, under some banner of party; when private friendships are violated and public confidence lost, it is cheering to behold a band of intelligent and honorable men uniting together in the peace and dignity of reason, for no sinister motive, but for the promotion of individual friendships and general harmony and benevolence.

"It was a fortunate idea, and reflects credit upon those who instituted and organized this society, that a nearer association and personal acquaintance among its learned members could but result favorably in producing and enhancing mutual confidence and esteem, while it would impart fresh zest to the pursuit of science.

"Similitude of interests and pursuits seems calculated to assimilate and bind together the enlightened and noble, and a generous rivalry but serves to enhance the ardor and enthusiasm of research. *Nil actum reputans, si quid superesset agendum.*

The medical profession, above all secular avocations, should most elevate and refine the mind, while it frees the heart from dross. Bringing its votary into immediate acquaintance with the secrets of the physical world, and not less so with the intricacies of the moral, it stamps him as the Priest of Nature.

"The physician stands upon a pedestal of exalted science, which should enable him to see beyond the conflicts of other men. Calm in reason and self-possessed, with a mind stored with virtue and religious contemplation, he is at once recognised as the friend of man and one of the noblest works of Deity.

"A generous and trusted friend in health, he becomes, in seasons of sickness, distress, and danger, a minister of mercy. True to himself, he has stored his mind with useful and varied knowledge, and now stands ready, with prompt alacrity and steady skill, to relieve or mitigate. Who has not felt, who can adequately appreciate the deep anxiety of the sufferer or still more suffering friends? The parent, wife, or child hangs with deepest solicitude upon his every look and slightest word. He is grave, and all is careful anxiety; he smiles, and cheerfulness returns with joy and buoyant hope.

"This is no 'fancy sketch' or dream of enthusiasm; the trail of the serpent is over the fairest flowers of human existence, and none are more frequently called upon to witness its withering blight.

"Nor is the benign influence of this noble profession bounded by States, Kingdoms, or parallels of latitude. Its reign is universal. Civilization confesses the obligation, and savage man looks on with wonder and veneration.

"Wherever the human race is found are its humanities represented, and ever and always ready to offer the balm and wine and oil of efficient sympathy, to console and assuage the distresses of our fellow-creatures.

"Well may we congratulate ourselves while surveying this brilliant field of our labors, more glorious than that of the 'cloth of gold;' and well may we exult in the words of the great Epic, '*Quæ regio in terris nostri non plena laboris.*'

"Gentlemen, we shall not detain you by a history of our profession, though it is ever gratifying to dwell upon the long list of mighty names which have in all times adorned its annals. These men and their deeds should be familiar to us as household words; and it only remains for us to imitate while we admire their virtues and their labors.

"Allow us, in conclusion, 'brethren of the mystic tie,' to assure you a cordial and honorable participation in the labors and privileges of the society; and while extending the right hand of fellowship, to wish you, all and each, in all sincerity, health and fraternity."—From *Dr. Page's Address* to the *Albemarle Med. and Philos. Society of North Carolina.*

